# Seroepidemiology of Helicobacter pylori infection among children seen in a tertiary hospital in Uyo, southern Nigeria

**Published:** 2012-06-21

**Authors:** Ofonime Michael Etukudo, Enobong Emmanuel Ikpeme, Emmanuel Eyo Ekanem

**Affiliations:** 1 Department of Paediatrics, University of Uyo Teaching Hospital, Uyo, Nigeria; 2 Department of Paediatrics, University of Calabar Teaching Hospital, Calabar, Calabar, Nigeria

**Keywords:** Helicobacter pylori, children, IgG seropositivity, epidemiology

## Abstract

**Background:**

Helicobacter pylori infection occurs worldwide with higher seroprevalence rates in the childhood populations of developing countries. In Nigeria, there is a dearth of information concerning its occurrence in children and infection enhancing factors. A prospective seroepidemiologic survey to determine the prevalence rate and possible associations of environmental and socio-demographic factors with its seropositivity was therefore conducted.

**Methods:**

The subjects were children seen at the Children's Emergency Unit of University of Uyo Teaching Hospital in southern Nigeria. Two hundred and thirty subjects, comprising 132(57.4%) males and 98(42.6%) females (male: female ratio= 1.3:1.0) with an age range of 0.5-15 years and a mean age of 5.0 (SD±4.0) years were recruited. The median age was 4.0 years. H. pylori immunoglobulin G (1gG) antibody was determined from serum samples stored at -200C using a commercial Enzyme-Linked Immunosorbent Assay (ELISA) kit, VicTorch.

**Results:**

The overall seroprevalence rate was 30.9% with a peak prevalence of 40.7% for the 6.0 to 10.0 years age group. H. pylori seroprevalence in our children is associated with low social class (p=0.038), increased household population (p=0.009), source of drinking water (p=0.014), type of convenience used (p=0.019) and the method of disposal of household waste (p=0.043).

**Conclusion:**

The seroprevalence of Helicobacter pylori infection in Nigerian children is high and is associated with low social class, poor domestic water and poor sanitation. Improvement of water supply, human and domestic waste disposal systems and ultimately poverty alleviation would control this bacterial infection that has severe long term consequences.

## Background

Helicobacter pylori, a curved spiral-shaped Gram negative bacterium, with a natural ecological niche at the antral portion of the human stomach has been associated with gastroduodenal diseases worldwide. It is estimated to be the commonest chronic human bacterial infection and about 50% of adults the world over are said to be infected with this pathogen [1, 2]. Manifestations of the gastroduodenal diseases are seen principally in adulthood although the acquisition occurs in childhood [3]. The highest incidence rates are recorded in the developing countries where over 50% of children are infected by five years of age [4–9]. Childhood acquisition in developed countries, though of a relatively lower prevalence, has also been reported [10–13].

Acquisition of infection in childhood reflects the social, environmental and economic status of the community [14]. Lower prevalence rates have been reported in communities which have a higher socioeconomic status and generally better environmental conditions [15].

The exact routes of H. pylori infection are still not known for certain. There are some supportive biologic epidemiologic evidences that transmission may occur through multiple pathways both from person to person and through external sources, with dominant routes perhaps varying between different populations. Person to person transmission is most commonly implicated with faeco-oral, oral-oral, or gastric-oral pathways [16].

Once the gastric mucosa has been colonised by the organism, there is a resultant chronic gastritis. H pylori gastritis is present in almost all adults and children with duodenal ulceration [17] but in absence of duodenal ulceration, it seems to be an asymptomatic infection in most, if not all, infected children [18–20].

H. pylori is ubiquitous but the prevalence, timing of acquisition and perhaps symptoms and the sequelae differ in developed compared to developing countries. There is need therefore for each community to determine its own prevalence of this global infection and make attempts to delineate the epidemiologic factors which may be associated with the infection, particularly during childhood. In Nigeria, most of the studies have been in adults, the only previous paediatric study was done about two decades ago [8]. This study was therefore designed to determine the seroprevalence of H. pylori infection in children attending a tertiary hospital in Nigeria and to see what epidemiologic factors may be associated with the seroprevalence.

## Methods

The study was conducted at the University of Uyo Teaching Hospital, Uyo, in Akwa Ibom State. Akwa Ibom State has a population of 3.9 million people. Uyo is the capital city of Akwa Ibom State. It is predominantly a civil service town. The Teaching Hospital is a 300 bed capacity hospital and the only tertiary health institution in the state.

The study was carried out between August 2008 and January 2009. The study population consisted of children between the ages of 6 months and 15 years who required venepuncture, and for whom an informed consent was obtained. A clinical history was obtained from each subject, including the family and social history and the child's socioeconomic status was determined using the parents/guardians social class according to the social classification scheme proposed by Oyedeji [21]. A quick clinical examination was also done including standard anthropometric measurements. These were recorded on the study proforma.

About 2.0mls of blood was collected from each subject into a plain specimen bottle and taken to the laboratory within 30 minutes and centrifuged to separate the serum, which was stored frozen at -200C till sufficient samples were pooled for analysis. The VicTorch H. pylori IgG test kit was used for the analysis according to the manufacturer's manual [22]. The reagents were stored unopened at 40C. Repeated freezing and thawing of the samples or microbial contamination, was avoided and icteric or turbid samples were not used (manufacturer's precautionary advice). The cut-off value for positive antibody activity (AA) was taken at greater than 20IU [22].

### Statistical analyses

Statistical analyses were performed using the SPSS software package (Statistical Package for Social Sciences) 14.0 Software. Data were summarised into tables. The seropositivity for H. pylori was computed according to age. Correlates of H. pylori were evaluated by a comparison of proportions of children with and without infection using Chi-square(x^2^) test and Fisher's exact test as appropriate. Controlling variables for evaluation with multiple logistic models were age, gender and social class.

The statistical significance of the adjusted seropositive rates among comparison groups was also tested. p-value of =0.05 was the significant level. Ethical approval was given by the Ethical Committee of the University of Uyo Teaching Hospital, Uyo.

## Results

Two hundred and thirty children aged 6 months to 15 years with mean age of 5.0 (±4.0) years, median age of 4.0 years, comprising 132 (57.4%) males and 98 (42.6%) females had their serum samples tested for IgG antibody reaction to H. pylori.

The age distributions of the study population are as shown in Table 1. Children less than 5 years of age constituted 60.0% while the 11- 15 years age group was the least represented (12.2%).

**Table 1:** Age distribution of the study population

Age (years)	Number	%	Cumulative%
< 1.0	30	13.0	13.0
1 – 5	108	47.0	60.0
6 – 10	64	27.8	87.8
11– 15	28	12.2	100.0

There were no gender differences in the general characteristics of the study population in terms of the mean age, weight, height and body mass index. The slightly higher values in males were not statistically significant. These characteristics are shown in Table 2.

**Table 2:** General characteristics of study population

	Gender		Total	p
	Male	Female		
Age (years) Mean (±SD)	5.2 (4.0)	5.2 (4.0)	5.2 (4.0)	0.910
Weight (kg) Mean (±SD)	18.7 (10.7)	18.5 (11.0)	18.7 (10.8)	0.846
Height (cm), Mean (±SD)	102.6 (28.0)	101.2 (28.7)	102.0 (28.3)	0.715
BMI (kg/m^2^), Mean (±SD)	16.8 (2.8)	16.4 (2.7)	16.6 (2.7)	0.217

Table 3 shows the distribution of the study population by age, social class and household population characteristics, including the number of siblings, and the number of persons sleeping on the same bed with the children. These were similar for both gender. A greater number of children were in social class III. More of them had two persons to share bed with, while one-sibling families and household populations of 4-6 members were more common. These values were however of no statistical significance.

**Table 3:** Distribution of study population by age, gender, social class and household population characteristics

	Male	Female	Both	*P*
	n (%)	n (%)	n (%)	
**Age (years)**				
< 1.0	18(13.6)	12(12.2)	30(13.0)	0.938
1 – 5	62(47.0)	46(46.9)	108(47.0)	
6 – 10	35(26.5)	29(29.6)	64(27.8)	
11 –15	17(60.7)	11(11.2)	28(12.2)	
**Social class**				
I	15(11.4)	11(11.3)	26(11.4)	0.643
II	32(24.2)	1919.6)	51(22.3)	
III	41(31.1)	31(32.0)	72(31.4)	
IV	35(26.5)	24(24.7)	59(25.8)	
V	9 (6.8)	12(12.4)	21(9.2)	
**No of persons in household**				
1 – 3	18(13.6)	16(16.3)	34(14.8)	0.299
4 – 6	76(57.6)	54(55.1)	130(56.5)	
7 – 9	31(23.5)	27(27.6)	58(25.2)	
10 – 12	7 (5.3)	1(1.0)	8(3.5)	
**No persons sleeping with child on the same bed**				
None	6 (4.5)	3(3.1)	9(3.9)	0.544
1	42(31.8)	40(40.8)	82(35.7)	
2	74(56.1)	48(49.0)	122(53.0)	
3 or more	10 (7.6)	7(7.1)	17(7.4)	
**Number of siblings**				
0	32(24.2)	24(25.5)	56(24.3)	0.401
1	18 (36.6)	21(21.4)	39(17.0)	
2	28 (21.2)	20(20.4)	48(20.9)	
3	26 (19.7)	29(20.4)	46(20.0)	
4	28 (21.2)	13(13.3)	41(17.8)	

Antibody activity (AA) of above 20IU was regarded as a positive reaction. Seventy-one subjects (30.9%) were serologically positive for H. pylori IgG antibodies while 159 (69.1%) were negative. The seropositive antibody activity (AA) level was >20.0IU with a mean (±SD) value of 42.0 (±16.1) compared with seronegative antibody activity of 0 to 20IU with a mean (±SD) value of 8.2 (6.1), p=0.000.

Table 4 shows that H. pylori seropositivity was slightly higher in the male subjects but this was of no statistical significance.

**Table 4:** Relationship between *H. pylori* seropositivity and gender of the subjects

Gender	*H. pylori* seropositivity (antibody activity (AA))	
	Positive (AA > 20)	Negative (AA ≤ 20)
	n (%)	n (%)
Male (n = 132)	46 (34.8)	86 (65.2)
Female (n = 98)	25 (25.5)	73 (74.5)
Total ( n = 230)	71 (30.9)	159 (69.1)

χ^2^ = 2.298, df = 1, *p* = 0.130

From Table 5, the H. pylori seropositivity was associated with the social class of the parents and the total number of persons in the household. However, it had no association with the number of siblings or the number of persons who sleep on the same bed with the subject. It also shows that the peak age prevalence of seropositivity was the 6.0 – 10.0 years age group. There was no statistical significance in the prevalence rates for the age groups.

**Table 5:** Relationship between *H. pylori* seropositivity and age, social status and characteristics of the household population of subjects

	Positive	Negative	All subjects	*p*
	n (%)	n (%)	n (%)	
**Age (years)**				
< 1.0	9 (30.0)	21 (70.0)	30 (100.0)	0.247
1.0 – 5.0	29(26.9)	79 (70.7)	108(100.0)	
6.0 – 10.0	26(40.9)	38 (59.4)	64 (100.0)	
11.0 – 15.0	7 (25.0)	21 (75.0)	28 (100.0)	
**Social class**				
I	8 (30.8)	18 (69.2)	26 (100.0)	0.038
II	11(21.6)	40 (78.4)	51 (100.0)	
III	19(26.4)	53 (73.6)	72 (100.0)	
IV	21(35.6)	38 (64.4)	59 (100.0)	
V	12(57.1)	9 (42.9)	21 (100.0)	
**No of persons in household**				
1 – 3	11(32.4)	23 (67.6)	34 (100.0)	0.009
4 – 6	30 23.1)	100(76.9)	130(100.0)	
7 – 9	25(43.1)	33 (56.9)	58 (100.0)	
10 – 12	5 (62.5)	3 (37.5)	8 (100.0)	
**No persons sleeping with child on the same bed**				
None	2 (22.2)	7 (77.8)	9 (100.0)	0.674
1	23(28.0)	59 (72.0)	82 (100.0)	
2	39(32.0)	83 (68.0)	122 (100.0)	
3	7 (41.2)	10 (58.8)	17 (100.0)	
**Number of siblings**				
0	15 (21.1)	41 (25.8)	56 (24.3)	0.093
1	12 (16.9)	27 (17.0)	39 (17.0)	
2	13 (18.3)	35 (22.0)	48 (20.9)	
3	11 (15.3)	35 (22.0)	46 (20.0)	
4	20 (28.2)	21 (13.2)	41 (17.8)	

Table 6 shows that H. pylori seropositivity was associated with domestic water supply, the type of convenience used and the method of domestic waste disposal utilised in the home. There was no association with keeping pets in the home.

**Table 6:** Relationship between *H. pylori* seropositivity and the household facilities of subjects

Facilities	*H. pylori*		All subjects	*p*
	Positive	Negative	n (%)	
	n (%)	n (%)		
**Type of convenience**				
Pit latrine	40 (38.8)	63(61.2)	103 (100.0)	0.019
Water closet	31 (24.4)	96(75.6)	127 (100.0)	
**Domestic waste disposal**				
Open space	24 (46.2)	28 (53.8)	52(100.0)	0.043
Burying	2 (50.0)	2 (50.0)	4(100.0)	
Open burning	20 (30.8)	45 (69.2)	65(100.0)	
Incinerator	0 (0.0)	1 (100.0)	1(100.0)	
Public waste bins	25 (23.1)	83 (76.9)	108 (100.0)	
**Domestic water source**				
Borehole/well	63 (29.0)	154 (71.0)	217 (100.0)	0.014
Pipe borne	5 (38.5)	8(61.5)	13 (100.0)	
**Animal pets in the house**				
Yes	9 (45.0)	11 (55.0)	20 (100.0)	0.152
No	62 (29.5)	148 (70.5)	210 (100.0)	
**Pets’ closeness (among 20 subjects with pets in the house)**				
Yes	1 (25.0)	3 (75.0)	4 (100.0)	1.000*
No	6 (37.5)	10 (62.5)	16 (100.0)	

* *P* – Fisher's exact test used

## Discussion

The seroprevalence rate of 30.9% obtained in this prospective observational study is high and suggests that H. pylori infection is significant in the paediatric age group of the study locality. This is consistent with high prevalence rates reported among children in other developing countries. In China, the seroprevalence rate of a childhood population of 7-14 years old school children was 40.9% [23]. Langat et al [24] reported a prevalence of 45.6% in children less than three years old in Kenya, eastern Africa. In contrast, studies done in the developed countries have reported low prevalence rates in their childhood populations. Malaty et al [25] reported a childhood seroprevalence rate of 19% in the United states of America. GranstrÖm et al [10] reported a seroprevalence rate of 13.6% in a cohort study of Swedish school children. The higher prevalence rates in developing countries are thought to be a consequence of the poor socioeconomic conditions prevalent in these countries [2].

The data also showed that H. pylori infection is acquired early in this study population, as 13.0% of infants less than one year old were seropositive. The highest age-specific prevalence of 47.0% was seen in the 6.0-10.0 years age group, while the lowest prevalence was seen in the 11.0 – 15.0 years age group. This trend contrasts with the reports of increasing prevalence rate with increasing age from some other studies [8, 23, 25].

Though the prevalence rate obtained in this study is high, when compared to an earlier study carried out on a paediatric population in the northern part of the country, it is much lower. Holcombe et al [8] in 1992 reported an age specific prevalence rate of 82% in Maiduguri; north eastern Nigerian children aged 5 – 10 years. In another recent study in the country, Ugwuja and Ugwu [26] in 2007, reported a lower prevalence rate of 11% among patients aged =20 years in Abakaliki, south-eastern Nigeria. These lower prevalence rates could be due to differences in the study populations, since the Maiduguri study was community-based while those of Abakaliki and Uyo were hospital-based studies. Again, the lower prevalence rate of this study may represent an actual decrease in the prevalence of H. pylori infection. Studies have shown a trend of decreasing prevalence over the past one to two decades in both adult and paediatric populations. In a paediatric seroepidemiologic survey in Guangzhou, southern China [27], the overall age standardization seroprevalence rate was reported to have dropped from 30.8% in 1993 to 19.4% in 2003, among children aged 1-5 years. This gave a decreasing seroprevalence level of 13.2% (p < 0.001). This trend of decreasing seroprevalence of H. pylori infection has been attributed to an improvement in their general living conditions. The comparatively lower prevalence rate obtained in this study in comparison with what was found in Maiduguri about 17 years ago may be due to a similar reason of improved living conditions over these past years. It may also reflect the comparatively higher standards of hygiene in southern Nigeria.

There was no gender difference in the seroprevalence rates in this study. This observation has been reported by many other authors [2, 4, 8, 10–12]. A contrasting scenario was however reported by Xu et al [28] where a significantly higher prevalence rate was reported in the male subjects but it was difficult to exclude bias in the subject selection for that study and that might have influenced the result.

The seroprevalence rate observed in this study showed a significant inverse association with the socioeconomic status of the parents. The study population is in a developing country and within the study population a significantly higher seroprevalence was noted with the lowest social class. The social class was derived from the occupation and educational attainment of parents. These observations lend support to the fact that low socioeconomic status impacts adversely on the prevalence of this infection. Low socioeconomic background and its natural consequences of overcrowding, poor hygiene and absent or insufficient sanitation have been reported to predispose to acquisition of H. pylori infection [29–31]. In a prospective population-based study of 1,545 Czech children aged 0 to 15 years, Sýkora et al [30] reported that the prevalence of H. pylori infection was higher in socioeconomically disadvantaged institutionalised children compared to healthy children living in family units. Socioeconomic variation is the major reason advanced to explain the relatively high prevalence rates in the developing compared to developed countries of the world [2, 10, 32]. A contrasting finding was however reported from a seroepidemiologic study of H. pylori infection among school children and teachers in Taiwan [33] where there was no association with socioeconomic status. This observation could have been due to subjects having similar socioeconomic background.

The seroprevalence of H. pylori was significantly associated with increased household population. Households with 10-12 members had almost two times the prevalence rate noted in smaller households of 1-3 members. This is consistent with the studies of Mendall et al [14], Brown [34] and Shi et al [29] which showed that domestic overcrowding in childhood is a risk factor for H. pylori infection.

The association with the source of domestic water supply, type of convenience used in the home and the methods utilised for the disposal of domestic waste, was also strong. The sources of drinking water used were mostly borehole and well water and this showed a higher risk of infection of H. pylori compared to pipe borne water. Also, use of pit latrine for faecal disposal was more risky than use of water closet toilet system. Open space disposal of household waste had a higher risk prediction than the public waste bin method. Several authors [29–31, 35, 36] have associated high prevalence of H. pylori with poor sanitation, especially poor faecal disposal and contact with faecal matter. Considering drinking water, Bode et al [19] reported a significant seropositivity with drinking of non-municipal sources of water among pre-school aged children in Germany. It has also been reported that molecular methods have detected the presence of H. pylori DNA in river water, well water, as well as surface or shallow ground water [37, 38]. An analysis of surface water and groundwater samples was done in Pennsylvania and Ohio. A microscopic technique that detected actively respiring microorganisms bound to monoclonal anti-H. pylori antibody was used. It was found that 61% of the samples were contaminated with H. pylori [37]. In contrast, a prospective cross-sectional epidemiologic survey of Czech children reported that domestic water sources and sanitary conditions had no association with the prevalence of H. pylori [30]. This contrary observation could have been due to their sanitary conditions being uniformly of good standard as all the homes had hot water and inside toilet facilities.

The lack of association with the number of siblings and number of persons sharing the same bed with the child, despite an association with increased total household population, was also observed by Shi et al [39] in a seroepidemiologic study in China. This finding may suggest that other factors of close family contact, and not necessarily sleeping together, may be responsible for the association of high prevalence of this infection with increased household population. For instance, Brown [34] reported that the culture of sharing eating plates within the household and premastication of food for infant were found to be independent risk factors for this infection in children.

## Conclusion

In conclusion, the prevalence of H. pylori infection is 30.9%. This is high among children in this southern Nigerian community. Infection also is acquired at very early age. Low socioeconomic status, poor human and domestic waste disposal systems and household crowding are some of the factors that enhance the infection. Obviously, the alleviation of poverty would help control the infection. This is a long term task. Meanwhile, improved water supply, human and domestic waste disposal systems could be rapidly implemented to control this bacterial infection that has severe long term consequences.
